# A Framework for Maternal Physical Activities and Health Monitoring Using Wearable Sensors

**DOI:** 10.3390/s21154949

**Published:** 2021-07-21

**Authors:** Farman Ullah, Asif Iqbal, Sumbul Iqbal, Daehan Kwak, Hafeez Anwar, Ajmal Khan, Rehmat Ullah, Huma Siddique, Kyung-Sup Kwak

**Affiliations:** 1 Department of Electrical & Computer Engineering, COMSATS University Islamabad-Attock Campus, Punjab 43600, Pakistan; sp15-bee-037@ciit-attock.edu.pk (S.I.); hafeez.anwar@ciit-attock.edu.pk (H.A.); drajmal@ciit-attock.edu.pk (A.K.); sp17-ree-016@ciit-attock.edu.pk (H.S.); 2 Department of Information and Communication Engineering, Inha University, Incheon 22212, Korea; asifsoul@inha.ac.kr; 3 Department of Computer Science, Kean University, Union, NJ 07083, USA; dkwak@kean.edu; 4 Department of Computer Systems Engineering, University of Engineering & Technology, Peshawar 25000, Pakistan; rehmatkttk@nwfpuet.edu.pk

**Keywords:** maternal physical activity recognition, wearable sensors, human-centric computing, raspberry-PI, BLE

## Abstract

We propose a physical activity recognition and monitoring framework based on wearable sensors during maternity. A physical activity can either create or prevent health issues during a given stage of pregnancy depending on its intensity. Thus, it becomes very important to provide continuous feedback by recognizing a physical activity and its intensity. However, such continuous monitoring is very challenging during the whole period of maternity. In addition, maintaining a record of each physical activity, and the time for which it was performed, is also a non-trivial task. We aim at such problems by first recognizing a physical activity via the data of wearable sensors that are put on various parts of body. We avoid the use of smartphones for such task due to the inconvenience caused by wearing it for activities such as “eating”. In our proposed framework, a module worn on body consists of three sensors: a 3-axis accelerometer, 3-axis gyroscope, and temperature sensor. The time-series data from these sensors are sent to a Raspberry-PI via Bluetooth Low Energy (BLE). Various statistical measures (features) of this data are then calculated and represented in features vectors. These feature vectors are then used to train a supervised machine learning algorithm called classifier for the recognition of physical activity from the sensors data. Based on such recognition, the proposed framework sends a message to the care-taker in case of unfavorable situation. We evaluated a number of well-known classifiers on various features developed from overlapped and non-overlapped window size of time-series data. Our novel dataset consists of 10 physical activities performed by 61 subjects at various stages of maternity. On the current dataset, we achieve the highest recognition rate of 89% which is encouraging for a monitoring and feedback system.

## 1. Introduction

Physical activities are often instrumental in the enhancement of human physical and mental health. Their absence, on the other hand, can cause adverse effects on well-being such as obesity [1]. Specifically, they are influential during particular medical conditions such as gravidity, more commonly known as pregnancy. It is a unique period in a woman’s life where her lifestyle, behavior, and physical activeness can significantly affect her health, as well as that of her fetus [2]. It is shown that the physical activeness of a gravida improves both the maternal–fetal health [3,4,5,6,7] by avoiding adverse pregnancy complications and birth outcomes, such as pre-eclampsia, gestational diabetes, and preterm birth. Various standard health guidelines [8] exist around the world where moderate exercises are recommended during pregnancy with a special care about particular health conditions such as pre-eclampsia. However, during such conditions, certain physical activities are completely proscribed by the experts while others are limited [9,10]. A gravida (pregnant woman) should avoid physical activities such as prolong standing and all those carried out in high heat and humid environment. However, the lack of knowledge about the physical activities and the remoteness from a health specialist result in birth complications that prove life-threatening especially in developing countries [11].

This makes the close supervision and advice of experts important, which, if not taken into account, could result in serious health issues. Nonetheless, such supervision is often expensive and time-consuming due to the frequent and scheduled visits to the experts [12]. To overcome these difficulties, a system based on the expert knowledge can be incorporated that frequently monitors the physical activities of a gravida and gives its recommendations. In this paper, we aim at the development of such a system by remotely recognizing and monitoring the physical activities of a gravida via the data acquired from the wearable sensors worn by her. Our proposed system recognizes the physical activity and then sends its feedback such as the type of activity and the time period for which it was performed. In this way, the monitoring of physical activities becomes convenient both locally (for the gravida) and remotely (for the health supervisor).

**Figure 1:**
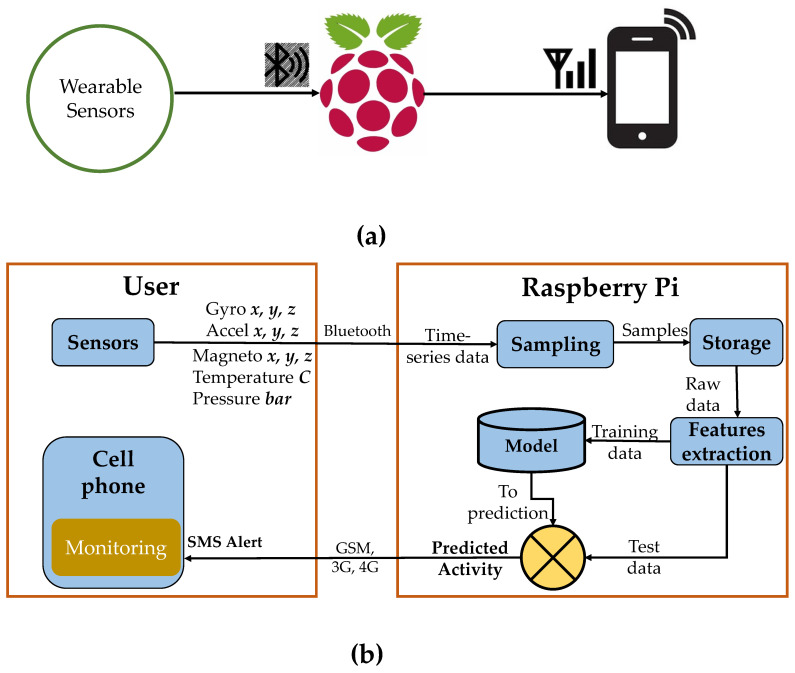
(**a**) Hardware and (**b**) software overview of the proposed framework.

Figure 1 shows an overview of the proposed system that involves several steps such as data acquisition, processing, recognition, and feedback. The data from gravida are acquired via wearable sensors modules that contain an accelerometer, a gyroscope, and temperature sensors. These data are then sent to a server such as a Raspberry-Pi via Bluetooth to perform the main step of physical activity recognition. Feedback is sent back to the user side about the recognized activity.

However, recognition of the physical activity based on the information of wearable sensors [13,14,15] is a non-trivial task that involves many challenges. For instance, the recognition rate is affected by the sensor placement on the body where a given physical activity may become more recognizable with sensors worn on particular body positions than others. The activities which include posture, bending, and ambulation moments are better monitored by placing sensors at hip, pocket, ankle, and thigh position, while activities involving upper body requires sensors to be placed at arm, chest, neck or elbow for better recognition. Similarly, the sensors installed at the pocket position can better recognize biking, stairs up and down while using the wrist position gives better recognition for eating and smoking [16]. The second challenging factor in wearable sensor-based activity recognition is the selection of useful features for a given activity. The first- and second-order statistics of the data extracted from various sensors, such as the accelerometer and gyroscope and their combinations, affect the recognition rates of the physical activities. However, some features boost the recognition rates of certain physical activities while degrading those of others. Finally, the selection of a machine learning algorithm to achieve the highest possible recognition rate is also challenging. A supervised machine learning algorithm that learns the model first such as random forest classifier gives a higher recognition rate given the trade-off that it is a tedious offline training procedure that requires a massive amount of data. On the other hand, the online feature matching algorithms such as the KNN classifier require no offline training procedure and data, but they tend to become time-consuming while matching a given physical activity with exemplar activities stored in the database.

In order to cope with these challenges, the following details our novel contributions in this paper.

1. As gravidity is a special body condition both medically and physically [17,18], we did not use the data of normal people to train the supervised machine learning algorithm for the activity recognition of gravidas. Instead, we collected a novel dataset of 10 physical activities from 61 gravidas who were at various stages of gravidity.
2. We performed features extraction on the acquired data using various statistical measures from both time and frequency domains.
3. For the physical activity recognition on the novel dataset, we evaluated several classifiers and selected the one that gave the best cumulative result.
4. We provide Raspberry-PI- and GSM-based real-time activities and health monitoring of gravida to avoid unfavorable situations in case of emergency.

The rest of paper is organized as follows. Section 2 provides the literature review. Section 4 explains the features engineering and classifiers. The dataset description, results, and performance evaluation are discussed in Section 5. Finally, we conclude the paper in Section 6.

## 2. Literature Review

In this section, we briefly introduce the prior contributions related to physical activities recognition (PAR) using wearable sensors. We summarize literature based on the following building blocks of a PAR system.

- Sensors used for the collection of data.
- Sensors placement on the body.
- Features vector construction from sensors data.
- Classification algorithms.

### 2.1. Sensors for PAR

PAR is mainly dependent on the raw data acquired through sensors that can be ambient, mobile, or wearable such as smartwatches. Ambient sensors are installed in the environment and have no physical connection with the person whose data is being acquired [19]. These sensors include video cameras [20,21], microphones [22], motion sensors [23], and depth cameras such as Kinect sensor [24], etc. These sensing systems are static and area-bound (Wi-Fi or Bluetooth range) where they can only monitor activities in the bounded area. For instance, if the gravida is working in an office, then she needs two separate sensing systems. In addition to that, the activities performed outside these two infrastructures cannot be monitored. For continuous data acquiring and monitoring, mobile and wearable sensors are used.

The smartphones [25,26] are equipped with multiple sensors such as an accelerometer, GPS trackers, Pulse sensor, gyroscope, etc. These sensors can acquire data remotely and accurately. However, the mobile sensor-based data acquisition system has some drawbacks [27]. Mostly, the smartphone is placed in a pocket position which reduces the accuracy of recognizing activities such as eating, typing, cooking, etc. Similarly, the continuous monitoring is also an issue with smartphones as they may stay away from the body in many cases, such as in handbags, for charging, lie on a table during office hours, etc. The body-worn sensors devices can avoid these issues and improve accuracy as they are continuous worn at various body positions. Consequently, a PAR system based on body worn sensors provides better results than those based on either ambient or mobile phone sensors [28,29].

### 2.2. Sensors Placement on Various Body Parts

The activity recognition accuracy highly depends on the sensors placement on various body parts. Performed physical activity can be better monitored by placing sensors on the body part that best suits the participant. The proper position of sensors solely depends on the activity being performed [30]. Furthermore, the literature shows that the PAR which include posture, bending, and ambulation moments are better monitored by placing sensors at hip, pocket, ankle, and thigh position, while activities involving upper body requires sensors to be placed at arm, chest, neck or elbow for proper recognition [31]. Table 1 shows the placement of body-worn devices on-body for various PAR.

### 2.3. Features Extraction from Sensors Data

In PAR, the sensors data are collected using various sampling frequencies according to the nature of acquiring activity. Liu et al. [42], as a first step, preprocessed the signal using a low-pass filter to remove the DC-component and then extracted features from the processed signal. They extracted a feature vector of length 24, including the mean, minimum, maximum, standard deviation, average peak frequency, root mean square, etc. The data are then segmented into a time-series segment known as windows size. Pannurat et al. [44] collected accelerometer data with 50 Hz and proposed a two-step process of feature extraction and selection. In the first step, they extracted 37 features using a window size of 1-s with 0.5-s overlap. In the second step, they used a Relief-F feature selection algorithm to select 7 features from the 37. Table 2 shows the literature summary of sensors, physical activities, the window size with overlapping and non-overlapping for features extraction, and corresponding extracted features. We adapted most of the features from the works in [45,46,47,48].

### 2.4. Classification Algorithms for PAR

Classifiers are supervised learning algorithms where the parameters of their respective models are trained using the training data samples along with labels. The recognition performance of this trained model is then evaluated by using it to predict the labels of completely new test data. Various classifiers are evaluated by different PAR systems such as K-nearest neighbor (KNN), decision tree, random forest (RF), gradient boosting, multilayer perceptron (MLP), artificial neural network (ANN), support vector machine (SVM), etc. [54]. Table 3 summarizes some methods with respect to the physical activities, classifiers, and their achieved recognition rates.

## 3. Data Set Description

**Figure 2:**
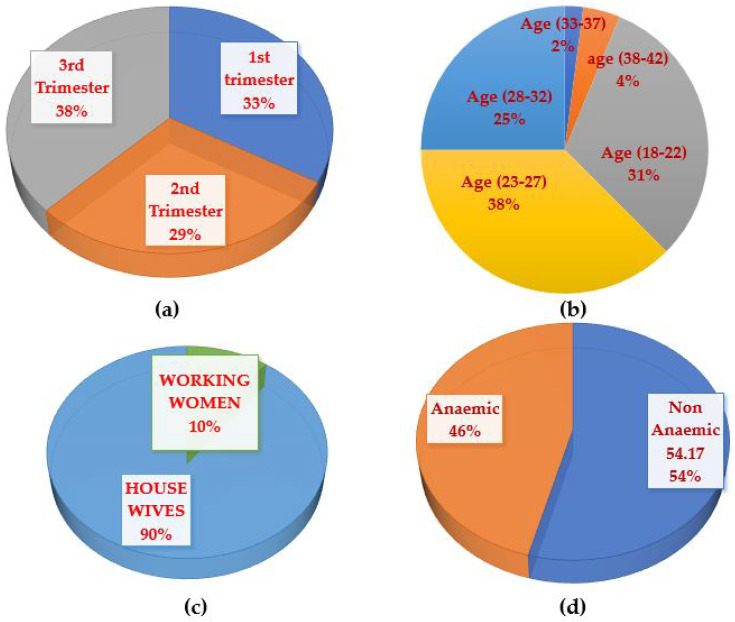
Maternal Patients Participants Distribution. (**a**) Trimester-based. (**b**) Age-based Distribution. (**c**) Profession-based. (**d**) Anemic and Non-anemic.

In this paper, we collect the data of pregnant women by installing the wearable sensor module at wrist position on either the left or right hand. We used the sensor placement at the wrist position because, mostly, the recognized activities involve hand movement. Sensor installation is easy at the wrist and can be managed as a smart watch by the maternal patient. We collect the data in a hospital under the supervision of a medical doctor (gynecologist). For data collection, we collected data from 61 subjects for ten physical activities that were mostly acquired from the literature of normal persons physical activities recognition. The activities are stairs up/down, cooking, eating, hands exercise, laundry, laying, walking, front bending, side bending, and standing. Figure 2 shows the distribution of maternal patients according to trimester, age, occupation, and anemia status. The participants performed each activity for 2–5 min according the physical condition of gravida and gynecologist suggestion. The sensors tag (wearable sensor module) was installed either on left or right wrist of the participant. The data were collected in hospital and at home so some of the activities were not performed by each participant. An average six activities were performed by each participant.

## 4. The Proposed Maternal Physical Activities Recognition (MPAR) Framework

**Figure 3:**
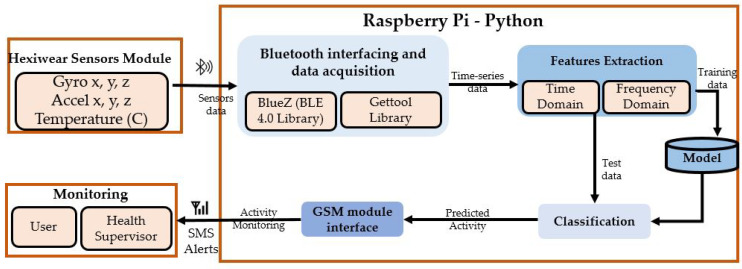
The proposed maternal physical activities recognition system architecture.

The goal of our proposed MPAR system is to collect data from sensors worn by a gravida, recognize her physical activities via these data, and send the monitoring messages to a health supervisor. Figure 3 depicts the complete architecture of our proposed MPAR system which consists of the following main modules:

1. Sensors module and data acquisition
2. Sampling and features extraction
3. Activity recognition
4. Monitoring

In the following, we give further explanation of each module.

### 4.1. Sensors Module and Data Acquisition

We use a single wearable module of sensor that consists of an accelerometer, a gyroscope, and temperature sensors installed at the wrist position. Table 4 shows the configuration of sensors in terms of sampling and quantization. Both the accelerometer and gyroscope have three dimensions: *x*-axis, *y*-axis, and *z*-axis.

The sensor module sends the data to a Raspberry PI using BLE 4.0 that has a communication range comparable to that of WiFi with an advantage of consuming almost 70% less energy during transmission. This leads to low battery power consumption of the sensor module [59]. On the software side, the connection between the Bluetooth BLE4 and Raspberry-PI is established using Python-based *BlueZ* while *Gettool* library is used for acquiring sensors data on Raspberry-PI [60].

### 4.2. Sampling and Features Extraction

**Figure 4:**
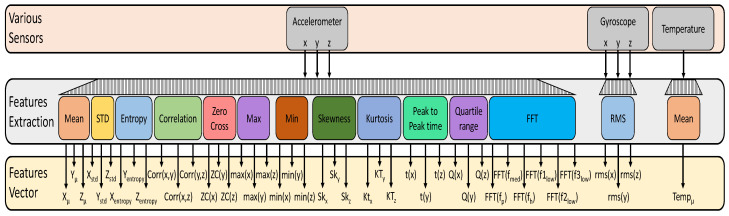
Feature vector construction.

We acquired the sensor data using various sampling frequencies and a sliding window approach where the two consecutive windows are either overlapping or non-overlapping. For instance, if the window size is one second, we get segments of the sensor data that are one second long. If the two consecutive windows become 50% overlapped, then their resultant segments will share 50% data. However, this is not the case in non-overlapping consecutive windows where the two segments do not share any data. We evaluate two values of window size which are one and two seconds. We use a 50% overlap, which means a 0.5 s overlap in consecutive windows of 1 s and a 1 s overlap in consecutive windows of 2 s. Each individual feature is extracted using 50 and 100 samples of accelerometer and gyroscope for 1 s and 2 s sliding windowing, respectively. We use 43 features to represent the sensors data that are shown graphically in listed in Figure 4 while their definitions are summarized in Table 5.

### 4.3. Activity Recognition via Supervised Machine Learning (Classification)

We evaluated a number of classifiers using WEKA [61] for our proposed MPAR system. Each classifier follows the general rule of supervised machine learning algorithms, where the classifier parameters are trained with the help of a training set and then its classification/recognition performance is evaluated with a completely disjoint test set. Our dataset consists of 10 physical activities represented via their feature vectors as shown previously. This dataset needs to be split into two disjoint training and test sets. However, for achieving the best recognition rate, the dataset is randomly split in training and test sets with various percentages of 90–10, 80–20, and 70–30. Consequently, experiments for each split are repeated multiple times and an average physical activity recognition rate is calculated. Table 6 shows the WEKA parameters configuration of the evaluated classifiers.

### 4.4. Monitoring

Mostly, people do not access to the data networks at all times. However, the GSM coverage is available at every place in a country. In order to provide the remote monitoring of a pregnant woman in case of an emergency or unfavorable conditions occur, we interfaced a GSM SIM900 module through a serial port with Raspberry-PI. The module keeps the numbers of registered gynecologists and care takers. The Raspberry-PI-based system monitors the maternal physical activities of the maternal woman, and if she is performing an activity that is not recommended it will send a message to the registered numbers..

## 5. Results and Discussion

Table 7 shows the feature extracted records of activities data acquired from gravidas. We used two different window sizes, i.e., one second and two seconds, and two configurations, i.e., overlapping and non-overlapping. In one second window size, the 0.5 s time-series data are overlapped and in two seconds window size the one second time-series data are overlapped. In this section, we show the recognition rates achieved by varying the following parameters.

- Sampling window size (overlapped and non-overlapped)
- Train and test split
- Type of classifier

**Figure 5:**
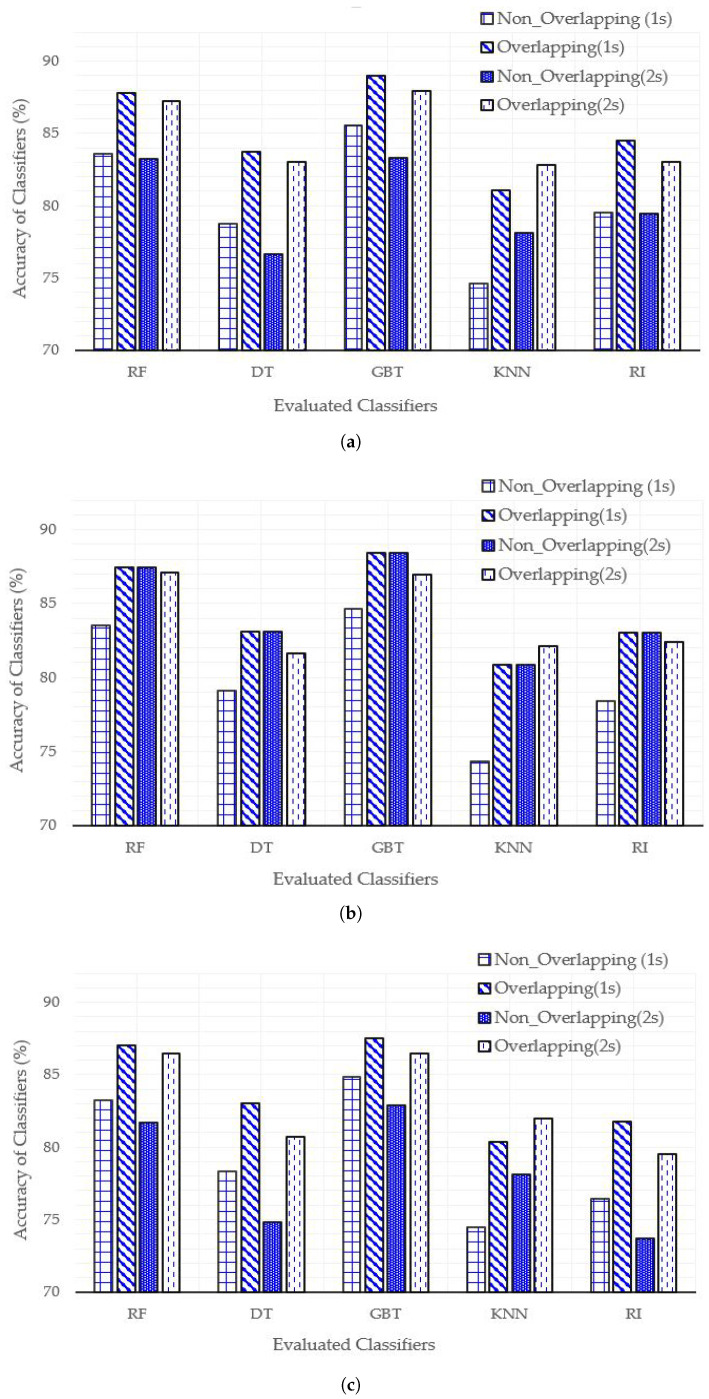
Classifiers performance comparison using various percentages of train–test splits: (**a**) 90–10, (**b**) 80–20, and (**c**) 70–30.

Figure 5 shows the results for all the above mentioned parameters. We summarize the main points of the achieved results in the following.

1. The sampling overlapped window of one second performs best in most of the cases except for KNN classifier.
2. The overall recognition rate of 80–20% data split is better than the other two.
3. The gradient boosted tree (GBT) classifier outperforms all the classifiers while KNN performs the worst of all.
4. The highest accuracy of 89% is achieved by GBT where the train–test split is 90–10% and sampling overlapped window size is one second.
5. The accuracy achieved by RF classifier is comparable to that of GBT in most of the cases. However, GBT has a higher computational complexity than RF, due to which we selected RF for the Raspberry Pi implementation.
6. The overall performance comparison depicts that the tree-based classifiers achieve higher accuracy than other classification algorithms.

Based on these observations, in the rest of this section, we shall explain the results achieved via RF classifier and with an overlapping sampling window of one second.

**Figure 6:**
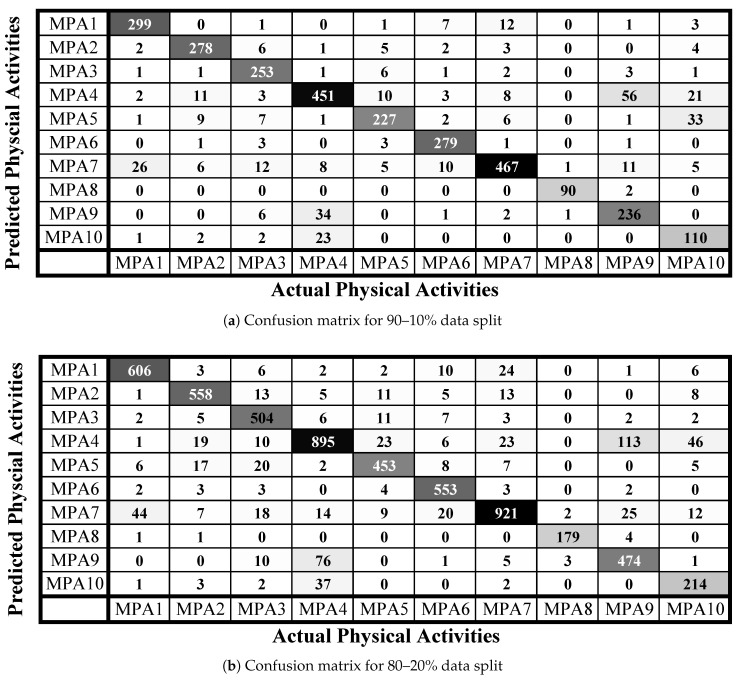

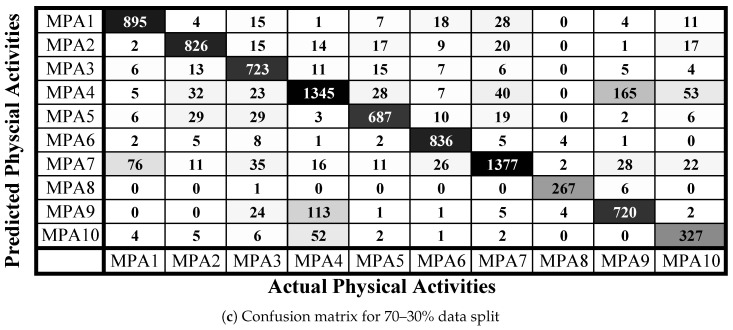
Confusion matrices achieved with a RF classifier where the training and test sets are split in various ratios.

Figure 6 shows the confusion matrices of individual activity recognition using all the three settings of dataset split. The following are the main observations.

1. The most recognized activity is front bending (MPA8), which is one of the most affecting physical activity during pregnancy. The best recognition rate achieved for this activity is almost 98% with the dataset split of 90–10.
2. The least recognized activity is standing (MPA10) which is also the least important among the current list of activities.
3. Side bending (MPA9) has greater confusion with the hand exercise (MPA4). Similarly, the standing activity (MPA10) has greater confusion with the hand exercise (MPA4). This is because the sensor module is installed at one hand wrist position and in all three activities there is little variation of linear and angular moments to perform these three activities.
4. Stairs walk (up/down) (MPA1) is confused with walking (MPA7), which is very convincing because during pregnancy climbing up or down the stairs is done with extreme care. This makes the stair walk very similar to the normal walk and consequently, the classifier confuses them for most of the times.
5. The physical activities where recognition rate is either equal to or higher than 90% are stairs Up/down (MPA1), cooking (MPA2), laying (MPA6), walking (MPA7), and front bending (MPA8).
6. All other activities except for side bending (MPA9) and standing (MPA10) are recognized with a rate of more than 85%.

## 6. Conclusions and Future Works

In this paper, we proposed a platform for maternal physical activities recognition using wearable sensors. The proposed architecture consists of a wearable sensors module that acquires the activity time-series sensory data and sends them to a Raspberry-PI based processing platform to extract the features and recognize the activity. We evaluated the window sizes with overlapping and non-overlapping to evaluate the performance of acquired maternal data and proposed platform. The experimental results showed that the window size of one second with overlapping technique perform better on all classification algorithms. The maternal physical activities data of ten activities are collected of 61 pregnant women. Five classification algorithms are evaluated to find the better algorithm that can be implemented on a real-time platform for maternal activities recognition. Overall, the proposed system recognize the activity with higher accuracy of 89%, which is encouraging, effective, and reliable.

In future work, we are planning to investigate the main aspects. The first aspect is considering the deep convolutional neural network for automatic features extraction and recognition. On the other hand, we want to expand the dataset by incorporating more activities and a larger number of participants. Furthermore, we are working on fusing sensors including breathing and ECG sensors, and sensors installed at multiple locations of body simultaneously.

## Figures and Tables

**Table 1:** Body-worn devices placement on-body for PAR.

S. No	Body-Worn Placement	Refs.
1.	Head	[32,33]
2.	Ear	[34]
3.	Shoulder	[35]
4.	Chest	[32,33,35,36]
5.	Arm (Elbow)	[32,36]
6.	Wrist	[32,37,38,39]
7.	Waist(Hip)	[32,38,40,41,42]
8.	Ankle	[34,37,38,40]
9.	Foot	[40]
10.	Knee	[34,43]
11.	Thigh	[32,33,38]
12.	Back	[35,43]

**Table 2:** Literature summary of sensors, physical activities, features extraction based on overlapping and non-overlapping, and selected features for HPAR.

Refs.	Sensors	Activities	OL/NOL	Features Extracted
[45]	Accelerometer, Gyroscope, and Magnetometer	PA1, PA7, PA10, PA11, PA26	NOL	Mean, Variance, Kurtosis Skewness Energy, Peak Signal Value, Maximum Latency, Peak-to-Peak Time, Peak-to-Peak Slope, Latency, Amplitude Ratio, Max. Amplitude, Min. Amplitude
[46]	accelerometer, gyroscope, magnetometer, quaternion	33-activities:PA7, PA26, PA15, etc.	(66%) OL	Mean, STD, median absolute deviation, maximum and minimum value, signal magnitude area, coefficients of auto-regression, index of frequency with largest coefficient, values of frequency kurtosis and skewness etc.
[49]	Accelerometer	PA1, PA7, PA10, PA11, PA12	(50%) OL, NOL	Mean, Binned range, Standard deviation, Time interval between local peaks, Correlation, Mean-dominant frequency, Mean energy of frequency
[50]	Accelerometer, Gyroscope	PA29 (Hand and Body Gestures)	(10, 30, 50, 60, 80)% OL	Convolutional Neural Network based Features
[51]	Accelerometer	PA1, PA7, PA10, PA11, PA24	(50%) OL	mean value, standard deviation, median absolute deviation, largest value, smallest value, signal magnitude area, energy, interquartile range, entropy, auto regression coefficients
[52]	Accelerometer, Gyroscope	PA30, PA31, PA32, PA33, PA34, PA35	(25%) OL	Entropy, Average, Standard Deviation, Number of Peaks , Number of Valleys
[53]	Accelerometer, gyro, Magneto, Heart rate, ECG	PA6, PA10, PA11, PA7, PA8, PA9, PA21, PA36, PA37, PA29	(Variable size) OL	code-based features approach

**Activities:** PA1: Stairs up/down, PA2: Cooking, PA3: Eating, PA4: Exercise, PA5: Laundry, PA6: Lying, PA7: Walking, PA8: Front-bending, PA9: Side-bending, PA10: Standing, PA11: Sitting, PA12: Jogging, PA13: Playing (games), PA14: Reading, PA15: Cycling, PA16: Gardening, PA17: Rope skipping, PA18: Rowing, PA19: Driving car, PA20: Uphill/ downhill, PA21: Stand-to-Sit, Sit-to-Stand, PA22: Sit-to-Lie, Lie-to-Sit, PA23: Stand to-Lie, Lie-to-Stand, PA24: Biking, PA25: Knee-bending, PA26: Running, PA27: Jumping, PA28: Washing dishes, PA29: Other activities, PA30: Brushing Teeth, PA31: Comb Hair, PA32: Drinking, PA33: Scratch Chin, PA34: Take Meds, P3A5:Wash Hands, PA36: Front Elevation of Arms, PA37: Stretching of Hands, PA38: Office Work.

**Table 3:** Literature summary of classifiers performance comparison for various physical activities.

Refs.	Physical Activities	Classifiers Performance Using Various Metrics
[54]	P29-(8 different activities)	SVM: 99.5%, Decision tree: 91.3%
[55]	PA1, PA2, PA14, PA28, PA7, PA13, etc.	Overall: 95%, DT: 83%, SVM: 84%, RF: 95%, ANN: 96%
[56]	PA1, PA27, PA6, PA12, P7, PA10	ANN: 77%, KNN: 75%, RF: 89%, SVM: 78%
[57]	PA1, PA6, PA7, PA10, PA11, PA15, PA26, *…*	About 87 ± 5% for decision tree, SVM, decision rules, KNN, Naive bayes, and RF
[58]	PA1, PA6, PA7, PA10, PA11	RF: 80%, MLP: 81%

**Table 4:** Sensors configuration (sampling and quantization).

Sensor	Sampling Frequency (Samples/Second)	Quantization Level
Gyroscope (x,y,z)	50	16-bit
Accelerometer (x,y,z)	50	16-bit
Temperature	1	16-bit

**Table 5:** Feature extraction from sensor data.

Features Name (Number)	Equations	Description
Mean (4)	$\mu =\frac{1}{N}\sum_{i=1}^{N}Sx_{i}$ where *N* are total samples equal to the window size and $S_{i}$ is a sample point of sensor data	Mean/Average value calculation for 3D Accelerometer (x,y,z) and temperature used to different between slow moving and fast moving activities.
Standard Deviation (3)	$\sigma =\sqrt{\frac{1}{N}\sum_{i=1}^{N}{\left(Sx_{i}-\mu \right)}^{2}}$	Find the spread in the accelerometer (x,y,z) data around their mean.
Cosine Similarity (3)	$Cos\theta =\frac{Sx.*Sy}{\left||Sx||||Sy|\right|}$ where Sx and Sy are the samples of accelerometer of *x* and *y*, respectively.	Find the cross-correlation in term of cosine similarity to differentiate between activities varying along with axis such as walking and stairs up/down. It is calculated between accelerometers *x* and *y*, *x* and *z*, and *y* and *z*.
Root Mean Square (RMS) (3)	$RMS_{x}=\sqrt{\frac{1}{N}\sum_{i=1}^{N}Gx_{i}}$ where $Gx_{i}$ is sample of x-axis gyroscope.	Find the angular movement along *x*-axis, y-axis, and *z*-axis, respectively. The RMS is calculated for the gyroscope sensors only.
Skewness (3)	$Sk_{x}=\frac{\sum_{i=1}^{N}{\left(Sx_{i}-\mu \right)}^{3}}{\sigma_{x}^{3}}$	Skewness measures the degree of symmetry in the accelerometer data distribution.
Kurtosis (3)	$Kt_{x}=\frac{\sum_{i=1}^{N}{\left(Sx_{i}-\mu \right)}^{4}}{N\sigma^{4}}$	Kurtosis measures the degree of tailedness in the accelerometer data distribution.
Max value (3)	$Accx_{max}=max\left\{Sx_{i}\right\}$	Calculate the maximum value of accelerometer (x,y,z)
Min value (3)	$Accx_{min}=min\left\{Sx_{i}\right\}$	Calculate the minimum value of accelerometer (x,y,z)
Zero crossing (3)	$ZC=count\{\left(\left(Sx_{i}<0\right)\&\&\left(Sx_{i+1}>0\right)\right)||\left(Sx_{i}>0\right)\&\&\left(Sx_{i+1}<0\right))\}$	Zero-Crossing is the number of times the signals crosses zero and its sign is changed.we consider ZC for the accelerometer along three axes.
Frequency Domain Features (6)	$H\left(k\right)=\sum_{n=0}^{N-1}x\left(n\right)e^{-2j\pi (\frac{kn}{N})}$	In this paper, we consider six frequency domain features based on the Fast Fourier Transform (FFT) of acceleration data. The six features are the FFT magnitude: $peak_{f}$ , $low_{f1}$, $low_{f2}$,$low_{f3}$, $med_{f}$, and $high_{f}$.
Entropy (3)	$Entropy=\frac{-1}{N}\sum_{i=0}^{N-1}px_{i}logpx_{i}$	Used for differentiation between activities of static and dynamic nature.
Quartile Range (3)	$Q_{1}=l+\frac{h}{f}\left(\frac{N}{4}-C\right)$	We find the first quartile (Q1), and it is defined as the middle number between the smallest number and the median of the sample data.
Absolute Time Difference between Peaks (3)	$|t_{maxpeak}-t_{minpeak}|$	It is calculated by taking absolute difference of time instance of maximum and minimum peak.

**Table 6:** Classification algorithms used to evaluate the performance comparison.

Classifier Type	WEKA Configuration	Abbreviation
K-Nearest Neighbors [62]	K = 1, Distance = Manhattan	KNN
Decision Tree [48]	Tree max depth = 50	J48
Random Forest [63]	No. of tress = 100	RF
Induction rules [64]	ratio of data for growing and pruning = 0.95	IR
Gradient boosted trees [65]	No. of tress = 100, depth = 50	GBT

**Table 7:** Features Extracted Dataset Description using Different Window Size and Overlapping/Non-Overlapping.

Activity Name (Label)	Window Size = 1 & Non-Overlapping	Window Size = 1 & Overlapping	Window Size = 2 & Non-Overlapping	Window Size = 2 & Overlapping
Stairs Up/Down (MPA1)	2217	3321	1052	1663
Cooking (MPA2)	1850	3082	1018	1542
Eating (MPA3)	1945	2929	968	1465
Hands Exercise (MPA4)	2997	5187	1574	2521
Laundry (MPA5)	1703	2566	849	1284
Laying (MPA6)	2025	3050	1009	1526
Walking (MPA7)	3411	5077	1720	2501
Front Bending (MPA8)	612	922	305	462
Side Bending (MPA9)	1908	3107	1087	1556
Standing (MPA10)	857	1472	427	736

## Data Availability

The dataset is available with the first author and will be provided on request.
